# Plant-Soil Mediated Effects of Long-Term Warming on Soil Nematodes of Alpine Meadows on the Qinghai–Tibetan Plateau

**DOI:** 10.3390/biology11111596

**Published:** 2022-10-31

**Authors:** Yanfang Liu, Wenying Wang, Pan Liu, Huakun Zhou, Zhe Chen, Ji Suonan

**Affiliations:** 1 The College of Geography Sciences, Qinghai Normal University, Xining 810008, China; 2 School of Life Sciences, Qinghai Normal University, Xining 810008, China; 3 Key Laboratory of Restoration Ecology in Cold Region of Qinghai Province, Northwest Plateau Institute of Biology, Chinese Academy of Sciences, Xining 810008, China

**Keywords:** climate warming, plant diversity, soil nematode communities, Qinghai–Tibetan Plateau

## Abstract

**Simple Summary:**

Global warming causes disturbances to grasslands and has a wide range of impacts on biodiversity and ecosystem processes worldwide. Soil nematodes are good indicators of climate change and are considered to be one of the important driving forces for the succession of plant communities. Therefore, understanding the relationship between soil nematodes and aboveground communities is particularly important under long-term warming. We selected an alpine meadow on the Qinghai–Tibetan Plateau and conducted a long-term artificial warming experiment with five different gradients. We found that both plant and soil nematode community composition were affected by long-term warming. In addition, plant diversity and community composition profoundly affect the diversity of soil nematode communities, thus reflecting the dynamic processes and evolution of soil ecosystems.

**Abstract:**

Global warming is one of the most pressing environmental issues today. Our study aimed to investigate how warming affected plant and soil nematode communities in alpine meadows on the Qinghai–Tibetan Plateau over the past seven years. An artificial warming experiment with different gradients was conducted from 2011 to 2018, including temperature increases of 0 °C (CK), 0.53 °C (A), 1.15 °C (B), 2.07 °C (C), and 2.17 °C (D), respectively. Cyperaceae plants were shown to be eliminated by increasing temperature, and plant community composition tended to cluster differently under different warming gradients. The number of nematodes decreased with the increase in soil depth, and the majority of them were observed in the topsoil layer. The individual densities of soil nematodes were 197 ind.·100 g^−1^ dry soil at 10–20 cm and 188 ind.·100 g^−1^ dry soil at 20–30 cm in the A treatment, which was significantly higher than the CK (53 and 67 ind.·100 g^−1^ dry soil) (*p* < 0.05). The lowest relative abundance of bacterivore nematodes (Ba) was 31.31% in treatment A and reached the highest of 47.14% under the warming gradient of D (*p* < 0.05). The abundance of plant parasitic nematodes (Pp) was significantly reduced to 26.03% by excessive warming (2.17 °C increase) in comparison to CK (41.65%). The soil nematode community had the highest diversity with a 0.53 °C increase in soil temperature; 1.15 °C warming gradients were lower, and nematode communities tended to be simplified (*p* < 0.05). All nematode channel ratio (NCR) values were above 0.5, indicating that warming did not change the decomposition pathway of soil organic matter dominated by the bacterial channels. The Wasilewska Index (WI) in the D treatment increased significantly compared to other treatments (*p* < 0.05), indicating that the mineralized pathway of the food web was primarily involved with Ba and fungivores nematodes (Fu), which is conducive to the growth of micro-biophagous nematodes. The plant parasite index (PPI) decreased significantly in the D treatment compared with other treatments (*p* < 0.05), indicating that a high warming gradient caused a reduction in the maturity of Pp nematodes. The maturity index (MI) increased in the D treatment compared with A, B, and C treatments, indicating that overheating affected the nematode community in the later stage of succession and caused the soil to be less disturbed. A partial least squares path model (PLSPM) showed that warming indirectly affects Fu and Pp diversity by directly impacting the plant community as well as indirectly affecting Ba by directly affecting soil properties. In conclusion, plant diversity and community composition profoundly affect the soil nematode communities, thus reflecting the dynamic processes and evolution of soil ecosystems.

## 1. Introduction

Global warming causes disturbances to grasslands and has a wide range of impacts on ecosystem biodiversity and processes worldwide [1,2]. The degradation of grasslands over the past several decades has been severely affected by global warming and human activities such as excessive grazing and inappropriate planting techniques [3]. A key animal husbandry base ecological safety barrier, the Qinghai–Tibetan Plateau (QTP) is home to an abundance of alpine grassland resources covering more than ~1.5 × 10^6^ km^2^ area, constituting 37.64% of the total grassland area in China [4]. The QTP is essential for maintaining ecological balance, water conservation, regulating the climate, conserving species diversity, and encouraging economic growth [4,5]. The Qinghai–Tibetan Plateau is warming, which will change grassland ecosystem characteristics [6,7]. Presently, the research in this field focuses primarily on the biomass, diversity, and phenology of grassland plants [7]. Soil nematodes are sensitive to climate and environmental changes and become one of the indicator organisms of climate change [8]. Its underground is complex and diverse and plays a critical role in driving soil nutrient cycling and maintaining soil physical structure through synergy [9]. Belowground biodiversity regulates aboveground biodiversity and ecosystem functions and is a sign of ecosystem changes [10]. As a part of this ecosystem, how do soil nematodes respond to global warming? In order to determine the answer, long-term experimental studies are required to investigate how soil nematodes respond to global warming and how they adapt to it.

Soil nematodes constitute a dominant group in the soil fauna and a particularly active class of organisms in plant rhizosphere soils [11]. They are often regarded as principal consumers in grassland ecosystems and are closely related to grassland productivity [12]. Due to their intimate relationship with plants, soil microorganisms and other soil animals play a crucial role in ecological processes such as the transformation of nutrients and the decomposition of organic materials in the soil [13]. Compared with other soil fauna: (1) nematodes are the dominant group of soil fauna; (2) they are easily isolated, and their separation efficiency can reach 97–99%; (3) family and genus identification are relatively simple, and the analysis of community structure level has been used to assess soil health; (4) they live in soil interstitial water and can quickly reflect the changes of the soil microhabitat at a small scale; (5) they play an important role in the soil food web due to their diverse feeding habits [14]. They are the perfect bioindicators for belowground ecosystems due to their unique characteristics. Soil nematodes can also affect the ecosystem and food-web-related ecological functions [15,16]. Plant parasites can enhance photosynthetic carbon distribution to roots, increasing the activity of root exudates and microorganisms [17]. A close relationship exists between nutrient mineralization, bacterivores, and fungivores in the soil [18]. Omnivores/predators are secondary consumers, feeding on small animals and other nematodes in the soil [19]. Thus, the species diversity of nematodes is linked to a system and its succession state, providing an effective way to study the ecological function and role of soil nematode communities.

The principal factors affecting soil nematodes are climate warming-induced microhabitat changes in the soil, such as primary productivity, soil microbial biomass, food sources, and soil moisture [20,21]. In addition, nematode communities are associated with the ecological functions of soil ecosystems and warming gradients [22,23]. Warming has significantly reduced the population density of nematodes by altering the soil microenvironment [24]. Matute [25] found in farm studies that warming changed the abundance of soil nematodes. Studies have shown that warming increases plant transpiration, reduces soil water content, and changes the microenvironment of nematodes. At the same time, temperature increases plant root growth and increases substrate supply for respiration, thus affecting the soil nematode community. According to Dong et al. [26], the species diversity of soil nematode communities in farmland ecosystems increased significantly because of warming. However, these various findings came from research on different ecosystems and warming ranges. How soil nematodes respond to global warming in alpine grasslands is essential. QTP is extremely cold, particularly due to hypoxia and strong ultraviolet radiation, coupled with the vulnerability and sensitivity of the alpine grassland ecosystem [27]. It is highly susceptible to the impact of global climate change and human activities, resulting in sandstorms and desertification [27]. Alpine grasslands are more susceptible to natural and artificial disturbances than other varieties of grasslands. Past studies usually focused on plant and soil nematode responses to different disturbance patterns, ignoring the importance of community composition and function, soil food webs, and synergistic interactions [14,24,25,26]. Therefore, it is necessary to study the effects of climate warming on biodiversity, soil food webs, and their synergistic effects in alpine grasslands.

In this study, we conducted a long-term artificial gradient warming experiment over seven years in a Haibei alpine meadow in the QTP. We aimed to advance knowledge of how plant and soil nematodes’ diversity, abundance, community structure, and synergistic interactions respond to global warming. This study provides a solid scientific foundation for comprehending changes in the alpine ecosystem’s structure and ecological processes.

## 2. Materials and Methods

### 2.1. Site Description

**Figure 1:**
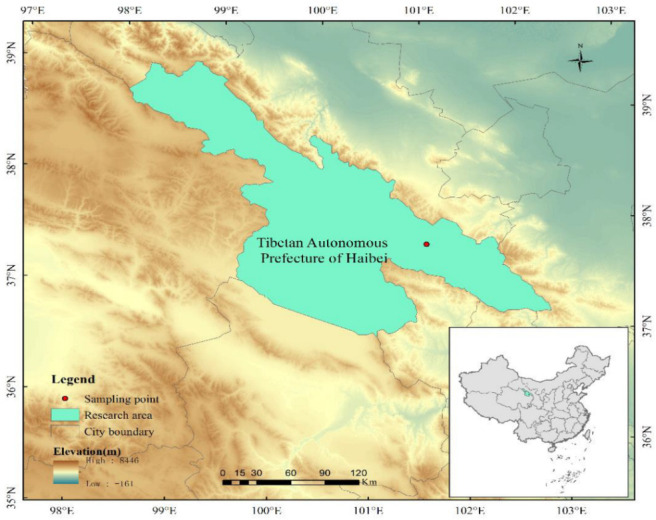
Research site (research Station of Haibei Boreal Meadow Ecosystem in the Qinghai–Tibetan Plateau, China).

The research site is located at the Haibei Alpine Meadow Ecosystem Open Experimental Station of the Chinese Academy of Sciences (hereinafter referred to as the Haibei Station) (Figure 1). The station is located at 37°29′ N–37°45′ N, 101°12′ E–101°23′ E, at an altitude of 3200–3600 m. The average annual temperature is −1.7 °C, and the annual average precipitation is 643 mm, with rainfall concentrated in May–September, accounting for 80% of the total annual rainfall. Dominant species and subdominant species of plants are *Kobresia humilis*, *Elymus nutans*, *Potentilla anserina*, *Poa crymophila*, *Carex atrofusca*, and *Kobresia tibetica*. The region has typical alpine meadow soil, with a tough grass layer 10–15 cm thick and thin, well-developed soil with high organic matter content and abundant reserves of total nitrogen, phosphorus, and potassium [28].

### 2.2. Experimental Design

**Figure 2:**
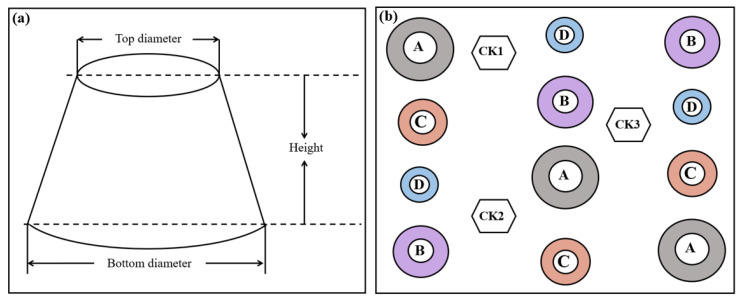
Experimental design for artificial climate warming experiment. (**a**): the shape of the OTC; (**b**): A–D represents OTC of different sizes, and CK is the control.

An open-top chamber (OTC) warming simulation experiment was conducted at Haibei Station in 2011. An artificial warming experiment with different gradients was conducted from 2011 to 2018. The study area was enclosed by a sturdy fence with a wide mesh, covering 50 m × 50 m for the sample plot. OTCs are constructed following the International Tundra Project’s consistent standards, using American-made glass fiber material (Sun-Lite^®^, Kalwall Corporation, Bow, NH, USA, HP; 1.0 mm thick) [29]. The OTCs were classified into four parts of different sizes (D, C, B, and A). The OTCs had bottom diameters of 1.15, 1.45, 1.75, and 2.05 m and top diameters of 0.70, 1.00, 1.30, and 1.60 m for groups D, C, B, and A, respectively. The soil temperature at 5 cm below the surface increased by 0.53 °C, 1.15 °C, 2.07 °C, and 2.17 °C in groups A through D, respectively. Therefore, we compared the effects of different warming gradients. There were five treatments, namely control (CK), warming 0.53 °C (A), 1.15 °C (B), 2.07 °C (C), and 2.17 °C (D), with 3 replicates in each group. Each individual sample plot was 1 m × 2 m (2 m^2^) in size. All plots were distributed randomly (Figure 2).

### 2.3. Plant and Soil Sampling

Three sampling points were randomly placed in each OTC during the growing period of the grassland in August 2018. Each plot had a 50 × 50 cm quadrat setup to assess the species coverage. All plants were divided into four functional groups, cyperaceae, legumes, grasses, and forbs, to count the individuals.

Surface vegetation was removed, and undisturbed soil samples were collected with a soil drill (5 cm diameter) from soil layers that were 0–5, 5–10, 10–20, 20–30, and 30–40 cm beneath the surface. Soil collected from the same layer was mixed in a polyethylene bag. The collected soil samples were separated into two parts, one part was stored at 4 °C for soil nematode analysis, and the other was naturally air-dried and sieved (pore diameters of 2 mm and 0.25 mm, respectively) for analysis of soil properties.

### 2.4. Determination of Soil Physical and Chemical Indexes

The physicochemical compositions of the soil were analyzed. The soil water content was measured using 10 g of soil that were dried in an oven at 105 °C for 12 h. The pH of a 1:5 air-dried soil:water suspension was determined using a pH meter. Available phosphorus (AP), ammonium nitrogen (NH_4_^+^-N), and nitrate nitrogen (NO_3_^−^-N) were measured using a FUTURA continuous flow analyzer (KPM Analytics, Estborough, MA, USA). Total inorganic nitrogen (IN) was calculated as the sum of NH_4_^+^-N and NO_3_^−^-N. Total carbon (TC) and total nitrogen (TN) were measured using an automatic carbon analyzer (Elementar, Langenselbold, Germany).

### 2.5. Soil Nematodes Extraction and Identification

A precise analytical balance was used (model: PTX-FA2105; measurement range: 210.0000 g) to weigh and record each fresh soil sample 50.00–70.00 g. The average mass was 60.00 g. Nematodes were separated over a 48 h period using an enhanced Baermann funnel method, and then their suspension was collected in a test tube [30]. Nematodes were counted using an inverted microscope, and their abundance was calculated using the amount of water content in 100 g of dry soil. The freshly collected nematodes were placed in a water bath at 62 °C for 3 min, then transferred to a 1.5 mL centrifuge tube with 1 × FG fixation solution (10:1:89 4% formaldehyde solution: glycerin: distilled water) to be fixed for more than one week. The glycerol–ethanol method [31] was then used to dehydrate the fixed nematodes, and permanent slides were prepared. The nematodes were classified and identified by genus level under the Zeiss microscope. At the same time, nematodes were categorized into four trophic categories based on their feeding behaviors and esophageal characteristics: plant parasites (Pp), omnivores/predators (Op), fungivores (Fu), and bacterivores (Ba) [32].

### 2.6. Ecological Indices of Soil Nematodes

The composition of soil nematodes and diversity of species were described with the Shannon–Wiener diversity index (*H*′): $H^{\prime }=-\sum_{i=1}^{S}n_{i}/N\times \ln\left(n_{i\;}/N\right)$, where *N* is the total number of individuals from all groups in the community, *S* is the number of groups, and *n_i_* is the number of individuals of group *i*. Pielou’s evenness index (*J*′): $J^{\prime }=H/\ln S$, where *H*′ is the Shannon–Weiner diversity index and *S* is the total number of species. Simpson dominant index (*λ*): $\lambda =\sum_{\left(\frac{n_{i}}{N}\right)}2\;$ [33], where *n_i_* is the number of individuals in group *I* and *N* is the total number of individuals in the community. *H*′, *J*′, and *λ* are often used to characterize the diversity of nematode communities; the higher the *H*′ and *J*′, the smaller the value of the *λ*, and the higher the species diversity of soil nematodes. The decomposition pathway of soil organic matter was exploited by the nematode channel ratio (NCR): NCR=Ba/(Ba+Fu) can be used to describe the importance of Ba and Fu in decomposition channels. When the NCR was higher than 0.5, it indicated that the decomposition of soil organic matter mainly depended on bacteria; on the contrary, it was dominated by fungal channels. The proportion between the decomposition and the primary production energy channels in the soil food web is described by the Wasilewska index (WI): WI=(Ba+Fu)/Pp. A low WI value indicates that soil nutrients and energy flow mostly from plants to herbivorous nematodes, whereas a high WI value indicates a larger activity of nutrient mineralization, with the involvement of bacterial and fungal-feeding nematodes [34]. The maturity index (MI) $\mathrm{MI}=\sum^{\;}c_{i}\times p_{i},$ is used to evaluate functional changes in a soil ecosystem after disturbance and restoration. Low MI indicates that the nematode community is in the early stage of succession and the soil is highly disturbed; whereas a high MI indicates that the nematode community is in the later stage of succession and the soil is less disturbed [32]; where *c_i_* is the colonizer-persister scale value (c-p) of the non-plant-parasitic soil nematode group *i*, and *P_i_* is the ratio of non-plant-parasitic soil nematodes (group *i*) to the total number of individuals in the soil nematode community. The plant parasite index (PPI), $\mathrm{PPI}=\sum^{\;}v_{i}\times f_{i},\;$ is a type of maturity index specifically targeted to plant-parasitic nematodes. PPI is closely related to primary production and is used to assess soil fertility [35]; where *c*(*i*) is the c-p value of the plant-parasitic nematode group *i*, and *P_i_* is the proportion of plant-parasitic individuals (group *i*) out of the total number of individuals in the soil nematode community. Subscripted numbers represent the c-p value for each species, assigned 1–5 according to different lifecycle strategies. For example, Ba_1_ represents a typical r-selected bacterial-feeding nematode (colonizer, early succession), whereas Op_5_ represents a typical k-selected omnivorous predator nematode (persister, late succession). The calculation methods used to determine the variables are b =$\;0.8\times \left(Pp_{2}+Fu_{2}\right)$, e = $\left(3.2\times Pp_{1}\right)+\left(0.8\times Fu_{2}\right)$, s = $\left(Ba_{n}\times W_{n}\right)+\left(Fu_{n}\times W_{n}\right)+\left(Op_{n}\times W_{n}\right)$, n = 3–5, W_3_ = 1.8, W_4_ = 3.2, and W_5_ = 5.0. The enrichment index (EI), $\mathrm{EI}=100\times \frac{e}{\left(e+b\right)},$ reflects the level of external nutrient input. A higher EI value represents more external nutrient input. EI is used to evaluate the response of the food web to available resources; Structure index (SI): $\mathrm{SI}=100\times \frac{S}{\left(S+b\right)},\;$ which represents the connectivity of the soil food web and the length of the food chain. The higher the SI value, the higher the relative connectivity and the longer the food chain. Both EI and SI are considered to assess soil enrichment and food web development [36].

### 2.7. Statistical Analysis

The effects of warming on soil physicochemical parameters, plant diversity, and soil nematode communities were examined using one-way analysis (ANOVA) and the least significant difference (LSD) through SPSS 19.0 (*p* < 0.05). The effects of warming on soil nematode density and distribution were examined using generalized linear models in SPSS with the 2-factor and interactions model (*p* < 0.05). Soil physicochemical properties used one layer of 0–40 cm data that was the average of all the soil layers. The 0–5, 5–10, 10–20, 20–30, and 30–40 cm values were used to calculate the density and dispersion of soil nematodes. Principal component analysis (PCA) was used to illustrate the relationships among plant functional groups under warming treatments, using CANOCO 5.0. Redundancy analysis (RDA) was performed with CANOCO 5.0 to analyze the relationships between soil nematodes and plant communities or soil properties. Graphics were created using GraphPad Prism 9.0. A partial least squares path modeling (PLSPM) was used to evaluate the relationship response between soil nutrients, plant communities, and nematodes using the R package “PLSPM” [37]. The model was evaluated using the goodness of fit (GOF); R^2^ values represent the proportion of each variable’s explained variance. RDA and PLSPM used one layer of 0–40 cm data that was the sum of all the layers.

## 3. Results

### 3.1. Soil Physicochemical Properties and Plant Species Diversity

**Figure 3:**
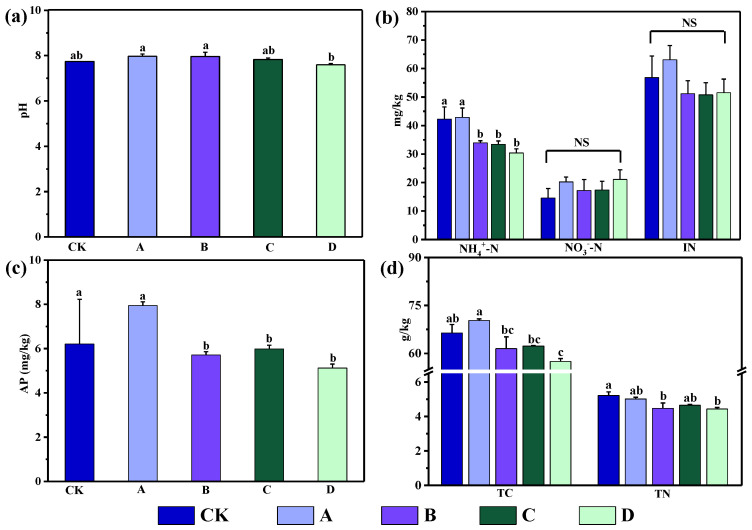
Soil physicochemical properties under different warming gradient treatments. (**a**) Soil pH; (**b**) ammonium nitrogen (NH_4_^+^-N), nitrate nitrogen (NO_3_^™^-N), and total inorganic nitrogen (IN); (**c**) available phosphorus (AP); (**d**) total carbon (TC) and total nitrogen (TN). Different lowercase letters indicate significant differences between treatments (*p* < 0.05); NS indicates no significant difference between treatments (*p* > 0.05).

The findings revealed that long-term warming raised soil pH, though the difference from CK was not statistically significant (Figure 3). In comparison to D, soil pH significantly increased under the A and B warming gradients (*p* < 0.05). CK and A treatment had significantly higher levels of ammonium nitrogen (NH_4_^+^-N) and available phosphorus (AP) compared to the other groups, while A treatment had the highest levels of both (*p* < 0.05). Total carbon (TC) concentration decreased compared with the CK and A treatment under the B, C, and D treatments, particularly under the D treatment. Total nitrogen (TN) significantly decreased in the C and D treatments compared with CK (*p* < 0.05). Gradient warming had no significant effect on the nitrate nitrogen (NO_3_^−^-N) or total inorganic nitrogen (IN) contents.

**Figure 4:**
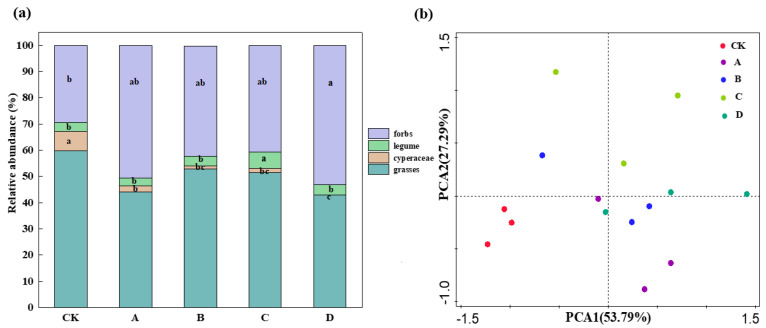
(**a**) Relative abundance of plant functional group composition and (**b**) principal component analysis (PCA) plot illustrating the distance between plant community composition under different warming gradient treatments. Different lowercase letters indicate significant differences between treatments (*p* < 0.05); the absence of letters means the difference is not significant (*p* > 0.05).

Warming produced different effects on the composition of the plant community (Figure 4a). The relative abundance of grasses decreased under warming treatment, although the difference was not statistically significant (*p* > 0.05). In contrast, the relative abundance of cyperaceae decreased significantly with increasing temperature (*p* < 0.05) and then disappeared entirely under the D temperature gradient. The relative abundance of legume plants under C treatment was significantly higher than others (*p* < 0.05). The relative abundance of weeds also increased along with temperature, with a significant difference in the D treatment compared to CK (*p* < 0.05; Figure 4a). Principal component analysis (PCA) results revealed that under various warming gradients, the composition of the plant communities tended to cluster individually (Figure 4b).

### 3.2. Soil Nematode Community Composition under Long-Term Gradient Warming

**Figure 5:**
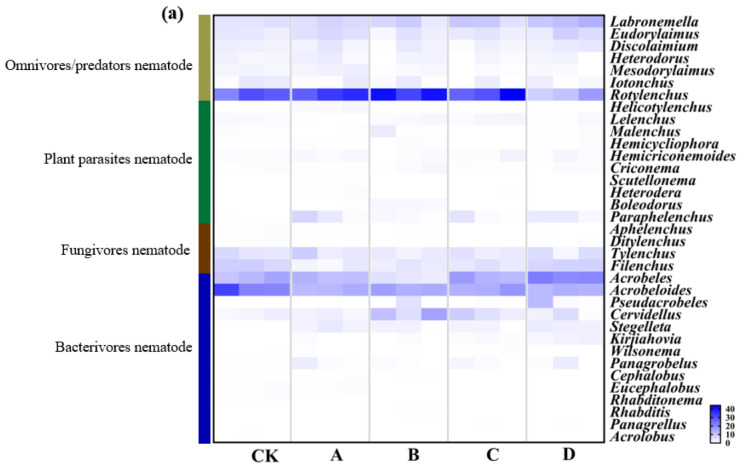

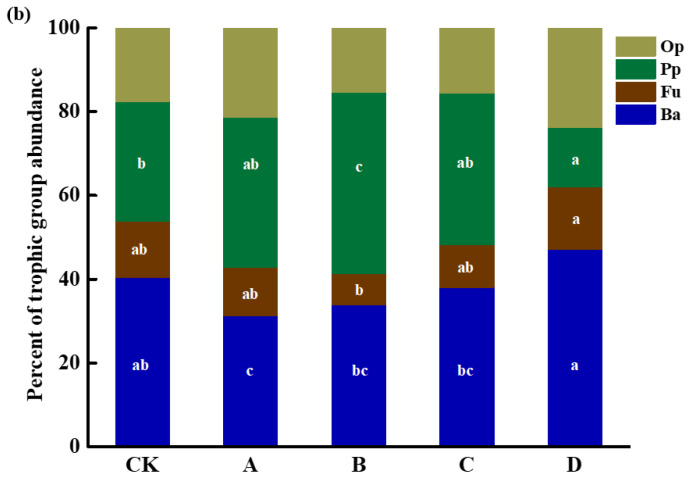
(**a**) Soil nematode community composition and (**b**) trophic group abundance under different warming gradient treatments. Ba, bacterivores; Fu, fungivores; Pp, plant parasites; Op, omnivores/predators. Different letters indicate significant differences between treatments (*p* < 0.05); the absence of letters means the difference is not significant (*p* > 0.05).

A total of 8882 nematodes were obtained from warming-treated soil samples belonging to 35 genera, 16 families, 6 orders, and 2 classes (Figure 5). The average individual density in dry soil was 1237 ind.·100 g^−1^. The dominant genera under the CK, A, and C treatments were *Acrobeloides* (Ba) (25.04%, 12.81%, 15.14%), *Acrobeles* (Ba) (12.59%, 11.68%, 14.08%), and *Rotylenchus* (Pp) (26.74%, 32.94%, 33.68%); under treatment B were *Rotylenchus* (Pp) (38.29%), *Acrobeloides* (Ba) (14.90%), and *Cervidellus* (Ba) (10.78%); whereas under treatment D they were *Labronemella* (Op) (11.40%), *Rotylenchus* (Pp) (11.93%), *Acrobeloides* (Ba) (12.95%), and *Acrobeles* (Ba) (20.92%) (Figure 5a). The relative abundance of Ba nematodes under five treatments was D (47.14%) > CK (40.31%) > C (38.00%) > B (33.80%) > A (31.31%) (Figure 5b). The abundance of Ba nematodes under the A temperature gradient decreased significantly (9.00%) (*p* < 0.05) compared to CK. There was a significant increase (15.83%) in the relative abundance of Ba in the D treatment with the temperature rise, compared with A (*p* < 0.05). The relative abundance of Fu nematodes first decreased and then increased under different warming gradients compared with CK (13.42%). Fu nematode’s relative abundance was significantly higher (14.91%) under the D warming gradient than 7.62% of the B treatment (*p* < 0.05). The relative abundance of Pp nematode in B (43.17%) and CK (28.72%) was significantly higher than that in the D treatment (14.10%) (*p* < 0.05).

### 3.3. Soil Nematode Density and Distribution under the Long-Term Gradient Warming

**Figure 6:**
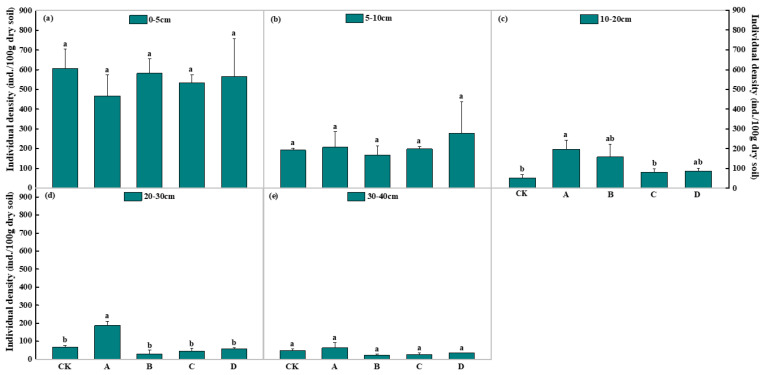
Soil nematode density and distribution under different warming gradients. (**a**) 0–5 cm, (**b**) 5–10 cm, (**c**) 10–20 cm, (**d**) 20–30 cm, and (**e**) 30–40 cm. Different letters indicate significant differences between treatments (*p* < 0.05); the same letter indicates no significant differences between treatments (*p* > 0.05).

One-way ANOVA showed that soil nematode density decreased with the increase in soil depth, mainly distributed in the 0–5 cm layer, and with manifest surface aggregation (*p* < 0.05) (Figure 6). In the A treatment, the individual densities of soil nematodes were 197 ind.·100 g^−1^ dry soil at 10–20 cm and 188 ind.·100 g^−1^ dry soil at 20–30 cm, which was significantly higher than the CK (53 and 67 ind.·100 g^−1^ dry soil) (*p* < 0.05; Figure 6). The individual density of nematodes in the 10–20 cm soil of the A treatment was also significantly higher than that of the C treatment at 80 ind.·100 g^−1^ dry soil (*p* < 0.05). However, there was no significant difference between CK, A, B, C, and D treatments at the depths of 0–5 cm, 5–10 cm, or 30–40 cm. The A treatment at 20–30 cm was significantly higher than the B, C, and D treatments, which contained 31, 45, and 59 ind.·100 g^−1^ dry soil, respectively. Two-way ANOVA showed that the soil layer had significant effects on nematode density (*p* < 0.01) (Table 1). The soil layer was the main factor affecting the variation of soil nematode density under the warming and soil layer.

### 3.4. The Response of Ecological Indices to Long-Term Warming

The soil nematode Shannon index (*H*′) and evenness index (*J*′) were both highest in the A warming treatment and lowest in the B group and significantly different in *J*′ between the two treatments (*p* < 0.05) (Table 2). The Simpson dominance index (λ) showed the reverse pattern, being highest for the B warming treatment and lowest for the A group (*p* < 0.05). *λ* can show the existence and characteristics of dominant populations; the smaller the value, the higher the diversity in the soil nematode community. As a result, our findings showed that the soil nematode community in the A warming gradients had the highest level of diversity. Soil nematode communities in the B treatment were lower than those in the other treatment, and nematode communities tended to be simplified. All NCR values were over 0.5, indicating that warming did not alter the soil organic matter’s predominant bacterial channel decomposition pathway. WI in the D treatment increased significantly compared to CK, A, B, and C (*p* < 0.05), indicating that the mineralized pathway of the food web was primarily involved with Ba and Fu nematodes, which is conducive to the growth of micro-biophagous nematodes. The soil food web mineralization was from plants to Pp nematodes because WI was lowest in B. PPI decreased significantly in the D treatment compared with other treatments (*p* < 0.05), indicating that a high warming gradient caused a reduction in the maturity of Pp nematodes. MI increased in the D treatment compared with A, B, and C treatments, indicating that overheating was caused in the nematode community in the later stage of succession, and the soil was less disturbed. The A warming gradient treatment consistently showed the highest EI and SI values. There was no significant difference among other treatments (*p* > 0.05).

### 3.5. The Relationship of Soil Properties and Plant Functional Groups with the Soil Nematode Communities

**Figure 7:**
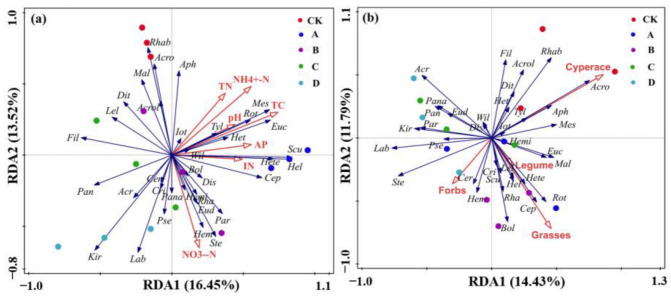
Redundancy analysis (RDA) describing the relationship between soil characteristics (**a**) and plant functional groups (**b**) and soil nematode communities under warming treatment. (*Acr*, *Acrobeles; Acro*, *Acrobeloides; Pse*, *Pseudacrobeles; Cer*, *Cervidellus; Rot*, *Rotylenchus; Hel*, *Helicotylenchus; Ste*, *Stegelleta; Kir*, *kirjiahovia; Wil*, *Wilsonema; Pana*, *Panagrobelus; Cep*, *Cephalobus; Euc*, *Eucephalobus; Par*, *Paraphelenchus; Aph*, *Aphelenchus; Tyl*, *Tylenchus; Fil*, *Filenchus; Lel*, *Lelenchus; Mal*, *Malenchus; Lab*, *Labronemella; Eud*, *Eudorylaimus; Dis*, *Discolaimium; Het*, *Heterodorus; Mes*, *Mesodorylaimus; Iot*, *Iotonchus; Hem*, *Hemicycliophora; Hemi*, *Hemicriconemoides; Cri*, *Criconema; Dit*, *Ditylenchus; Rhab*, *Rhabditonema; Scu*, *Scutellonema; Hete*, *Heterodera; Bol*, *Boleodorus; Rha*, *Rhabditis; Pan*, *Panagrellus; Acrol*, *Acrolobus Bol*, *Boleodorus*).

The effects of environmental factors and plant communities on soil nematode communities were analyzed using redundancy analysis (Figure 7). The first axis explained that 16.45% and 14.43% of the changes in the soil nematode community were influenced by soil variables and plant functional groups, respectively (R^2^ = 0.53). The soil nematode species varied by different warming treatments, suggesting that soil nematodes have great potential to indicate temperature change at the genus level (Figure 7a). The increased distances between the various treatments indicate significant differences in soil properties and plant communities (Figure 7). In the soil properties RDA biplot (Figure 7a), it can be observed that soil NO_3_^−^-N was negatively correlated with soil Ba nematodes under CK treatment and influenced Ba phyla, including *Rhabditonema*, *Acrobeloides*, and *Acrolobus*. Pp nematodes *Scutellonema*, *Heterodera*, and *Helicotylenchus*, were positively correlated with soil AP and IN. NO_3_^−^-N showed positive correlations with *Stegelleta* (Ba) and *Hemicycliophora* (Pp) under B treatment. Soil pH, NH_4_^+^-N, TC, and TN were positively correlated with Ba and Pp communities (including *Panagrellus* and *Acrobeles*) in the C and D treatments. These results indicated that soil C, N, and P were the main factors affecting the distribution of Ba and Pp but had little effect on Fu and Op (Figure 7a).

Concerning plant communities (Figure 7b) (R^2^ = 0.33), cyperaceae plants were positively correlated with Ba and Fu communities under CK treatment, including *Acrobeloides* and *Aphelenchus*. Forbs plants showed a positive correlation with *Cervidellus* (Ba) and a negative correlation with *Rhabditonema* (Ba) under A treatment. Legume and grass plants showed positive correlations with Ba of the genus *Cephalobus* and Pp of the genera *Helicotylenchus* and *Rotylenchus*. Legumes and grasses showed a negative correlation with *Acrobeles* (Ba) and *Wilsonema* (Ba) under the C and D treatments; Forbs plants showed a negative correlation with *Criconema* (Pp) and *Scutellonema* (Pp). According to these findings, grasses, legumes, and forbs were the primary factors influencing the distribution of Ba and Pp, while cyperaceae had a higher impact on Fu. Plant community structure had little effect on Op.

### 3.6. Relationship between Soil Properties, Plant Diversity and Soil Nematodes Communities

**Figure 8:**
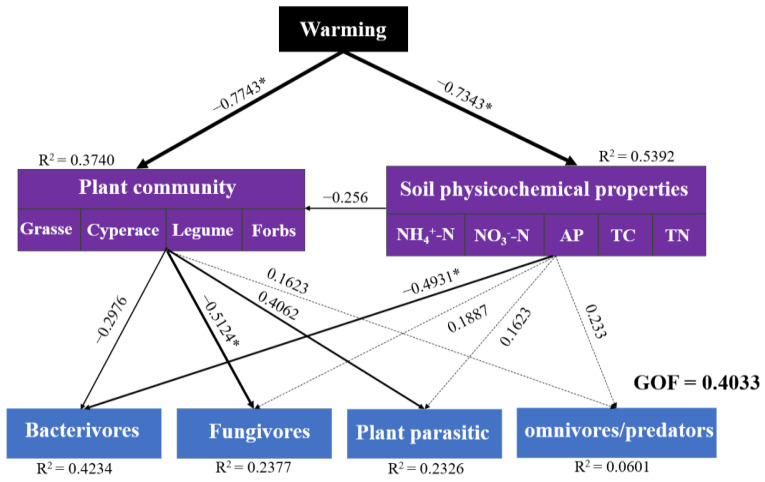
Partial least squares path modeling (PLSPM) for soil nutrients, plant communities, and nematode communities. Note: Bold and solid/dashed arrows indicate the strength and significance of pathway effects, respectively. The numbers above the arrows indicate path coefficients. The model was evaluated using goodness of fit (GOF). R^2^ values represent the proportion of each variable’s explained variance. Levels of significance: * *p* < 0.05.

The PLSPM indicated direct or indirect relationships between soil properties, plant communities, and nematodes (Figure 8). The model explained 37% and 54% of the plant and soil properties and explained 42%, 24%, 23%, and 6% of the variance in bacterivores, fungivores, plant parasites, and omnivores/predators, respectively. The plant community and soil properties were significantly impacted negatively by warming (*p* < 0.05). Plant diversity has a significant negative correlation with fungivores and a positive correlation with plant parasites (*p* < 0.05). Bacterivores and soil properties had a significant negative correlation (*p* < 0.05). In a nutshell, warming had an indirect effect on soil fungivore nematodes and plant parasite diversity by directly influencing the structure of plant communities. It also had an indirect impact on bacterivore nematodes by directly affecting the properties of the soil.

## 4. Discussion

### 4.1. The Impact of Long-Term Gradient Warming on Plant Communities

Previous research has demonstrated that warming significantly changes plant community structure and that the composition of plant communities tends to cluster separately, both of which are consistent with our results [38]. The environmental temperature was also usually lower than the optimum temperature required for plant growth in the hinterland of the QTP [39]. The physiological structures of plants and niches of different species are different. These differences, combined with the change in the warming ranges, lead to variations in the intensity of photosynthetic respiration, biomass distribution, and phenological phases of plants. This changes the composition of plant communities [40]. Here, we found that the significance of cyperaceae plants reduced significantly with a rise in warming temperature, with cyperaceae plants completely disappearing in the maximum temperature range. This may be due to rising temperatures exceeding the heat tolerance limit of cyperaceae plants, leading to the succession of plant communities and, thus, the reduction of species importance [40,41]. Results from Li et al. [41] relate to the effects of gradient warming on species diversity, and biomass in an alpine meadow is consistent with our results.

### 4.2. The Impact of Long-Term Warming on Soil Nematodes Communities

We identified soil nematodes in the Haibei alpine meadow, which are within the range of most prior reports [42]. Soil nematodes were most abundant in the 0–5 cm soil layer, and this topographical aggregation of quantity and genera was consistent with previous studies [43].

There were differences in the number of soil nematodes, community composition, and trophic group structure in the process of long-term warming. In this investigation, decreased warming raised the abundance of soil nematodes (the average 0.53 °C temperature increment). Temperature increases may have improved the microenvironment of soil nematode habitats and resource utilization, which increased soil nematode quantity [44]. Wang et al. [45] also found that increases in nematode numbers under warming treatment in alpine meadows of northern Tibet were caused by the adaptability of soil nematodes to temperature. Nematodes are aquatic animals that need soil-free water to perform their biological functions. As temperatures rise, soil moisture decreases, which decreases the number of nematodes [46]. Consistent with this, we found that soil water content decreased from 61.27% (CK) to 29.13% (D treatment) with the increase in temperature. Soil nematode abundance decreased under excessive warming (D treatment). Excessive warming increased transpiration, aggravating soil water loss and heat stress, which in turn led to an irreversible impact on the living space and reduced the number of soil nematodes [47]. During the process of long-term warming, various soil layers showed different responses from the soil nematode community. Soil nematode density decreased with the increase in soil depth, mainly distributed in the 0–5 cm layer, and with manifest surface aggregation [23,45,46]. This study’s results also indicated that the soil layer was the main factor affecting the variation of soil nematode density under warming and soil layers [23,47]. According to our findings, soil nematodes had a better chance of surviving in deeper soil layers under moderate warming disturbance. This phenomenon may relate to the buffering effect of the topsoil [23].

Trophic groups have different responses under increased temperatures. The nematode community was usually dominated by Ba and Pp, with low Op and Fu [48], which is consistent with our results. According to this study, the total abundance of Pp in the Haibei alpine meadow on the Qinghai–Tibet Plateau showed an increasing trend under moderate warming (0.53 °C increase). Legume and grass plants showed positive correlations with Pp of the genera *Helicotylenchus* and *Rotylenchus* with a 0.53 °C increase. This phenomenon demonstrated that moderate warming interference in alpine meadows leads to an increase in the abundance of Pp, making them more susceptible to Pp than normal alpine meadows [7,49]. Forbs plants showed a negative correlation with *Criconema* (Pp) and *Scutellonema* (Pp) under an increase of 2.17 °C. Excessive warming significantly reduced the abundance of Pp, indicating that high-gradient warming has an inhibitory effect on Pp [23,49]. Ba’s abundance first decreased significantly because of warming, then increased, indicating that warming had a major impact on Ba’s abundance, but Ba’s dominant position in the nematode community remained unaffected. This result is consistent with a previous study by Zhang et al. [23]. Xue et al. [49] studied three soil nematode communities in the alpine meadow in northern Tibet and found that the relative abundance of Ba was 25.25–42.82%, which is consistent with our results. The relatively high abundance of Ba may reflect an increase in bacteria and organic matter in the soil [50]. A specific warming temperature reduced the abundance of the Fu nematode (*p* < 0.05); however, with the increase in temperature, the Fu nematode adapted to warming, and the abundance increased. These results suggested that the alpine meadow system may show a trend of strengthening the food web driven by Fu under excessive long-term warming; previous studies have had similar results [51]. Op nematodes showed resistance to climate warming because their abundances were only slightly affected [52].

Nematodes are frequently employed as soil indicators to evaluate the biological effects of ecosystems and the degree of ecosystem succession or disturbance due to their distinct biological characteristics [53]. We found that the diversity and uniformity of nematode communities first increased, then decreased along with the increasing temperature, indicating that moderate warming would have beneficial effects on alpine ecosystems, consistent with the conclusions of Nielsen et al. [54] in tundra ecosystems. More community compositions and greater evenness are associated with increased community diversity, which can lead to the optimization of ecosystem function and the improvement of ecosystem stability [55]. Therefore, we speculate slightly higher temperatures will promote the stability of the soil ecosystem, whereas higher temperatures will inevitably result in the degradation of grasslands and a decline in ecosystem stability [21]. Ma et al.’s [56] results showed that soil warming temperature is enough to reach the threshold, which leads to significant changes in the MI of soil nematodes. Long-term overheating significant increased MI in this study. The stability of the ecosystem and the extent of a disturbance are frequently reflected in MI and PPI values [33]. MI increased in the 2.17 °C increase in the soil temperature, indicating that overheating was caused in the nematode community in the later stage of succession, and the soil was less disturbed [56]. PPI decreased significantly in the 2.17 °C increase, indicating that a high warming gradient caused a reduction in the maturity of Pp nematodes [35]. NCR indicated that the organic matter decomposition pathway of soil nematodes was not affected by warming, which was dominated by the bacterial path [57]. Based on EI and SI values, our results indicated that a moderate warming gradient might expand the food chain of soil nematodes, thus making a more complete nematode food web. A possible reason is that most of the nematode genera have better abilities for decomposition and mineralization with increased abundance in the moderate warming gradient.

## 5. Conclusions

Long-term gradient warming caused some differentiation in species composition and distribution and, thus, in functional groups of the plant and soil nematode communities. The results of principal component analysis (PCA) showed that the plant community composition tended to cluster separately under different warming gradients. Soil nematode density decreased with the increase in soil depth, mainly distributed in the 0–5 cm layer, with manifested surface aggregation. The soil layer was the main factor affecting the variation of soil nematode density under warming. Moderate warming (0.53 °C increase) improved the microenvironment of soil nematode habitats and resource utilization, which increased soil nematodes. The diversity indexes *H*′, *J*′, and λ and the functional group indexes PPI, MI, EI, and SI can be used as sensitive indicators to evaluate changes in soil nematode community structure. This reflects the changes in plant community and soil environment at 0 °C, 1.53 °C, and 2.17 °C, and is a quantitative index to evaluate the changes in soil nematode community structure. Partial least squares path modeling (PLSPM) showed that warming had an indirect effect on soil fungivores (Fu) and plant parasites (Pp) diversity by directly affecting plant communities; it also had an indirect effect on Ba by directly affecting soil properties.

## Figures and Tables

**Table 1:** Test of inter-subject effect of warming and soil depth for the soil nematode density.

Source	df	Mean Square	F	Sig.
Correction model	24	109,718.114	9.217	0.000
Intercept	1	2,846,987.035	239.159	0.000
Warming	4	6117.611	0.514	0.726
Soil layer	4	625,437.533	52.540	0.000 **
Warming * Soil layer	16	6688.384	0.562	0.897
Error	50	11,904.136		
Total	75			
Total corrected	74			

Note: R^2^ = 0.783, * *p* < 0.05, ** *p* < 0.01.

**Table 2:** Ecological index values of soil nematodes under warming treatments.

	CK	A	B	C	D
*H*′	2.15 ± 0.02 a	2.42 ± 0.10 a	2.1 ± 0.17 a	2.12 ± 0.14 a	2.31 ± 0.06 a
*J*′	0.70 ± 0.01 ab	0.78 ± 0.02 a	0.68 ± 0.03 b	0.72 ± 0.04 ab	0.72 ± 0.01 ab
*λ*	0.17 ± 0.00 ab	0.11 ± 0.01 b	0.20 ± 0.03 a	0.18 ± 0.03 a	0.16 ± 0.02 ab
MI	3.08 ± 0.05 ab	3.28 ± 0.05 b	3.07 ± 0.17 b	3.12 ± 0.16 b	3.98 ± 0.07 a
PPI	2.25 ± 0.08 a	2.38 ± 0.09 a	2.54 ± 0.10 a	2.47 ± 0.18 a	2.02 ± 0.10 b
NCR	0.75 ± 0.01 a	0.74 ± 0.04 a	0.80 ± 0.02 a	0.79 ± 0.02 a	0.76 ± 0.02 a
WI	1.93 ± 0.31 b	1.22 ± 0.23 b	1.02 ± 0.07 b	1.44 ± 0.34 b	5.11 ± 1.52 a
EI	40.56 ± 0.56 abc	44.81 ± 2.89 a	36.74 ± 0.38 c	42.86 ± 0.00 ab	39.72 ± 1.21 bc
SI	80.87 ± 0.81 a	84.31 ± 1.53 a	79.24 ± 1.59 a	82.6 ± 3.47 a	80.29 ± 2.06 a

Note: mean ± standard error. The same letter indicates no significant differences between warming treatments (*p* > 0.05), while different letters indicate significant differences between warming treatments (*p* < 0.05).

## Data Availability

Not applicable.
